# Exothermic properties of plaster–synthetic composite casts

**DOI:** 10.1007/s11832-014-0563-6

**Published:** 2014-02-20

**Authors:** Rolf D. Burghardt, John G. Anderson, Rob A. Reed, John E. Herzenberg

**Affiliations:** 1 International Center for Limb Lengthening, Rubin Institute for Advanced Orthopedics, Sinai Hospital, 2401 West Belvedere Avenue, Baltimore, MD 21215-5271 USA; 2 East Leonard Medical Complex, 1111 Leffingwell Ave Ste 100, Grand Rapids, MI 49525 USA; 3 Department of Radiology, Oakwood Hospital, 1800 Oakwood Blvd, Dearborn, MI 48124 USA; 4 Orthopaedic Department, Helios-Endo Klinik Hamburg, Holstenstraße, 22767 Hamburg, Germany

**Keywords:** Plaster of paris, Synthethic cast, Composite cast, Skin burn, Cast complication

## Abstract

**Purpose:**

Plaster casts can cause burns. Synthetic casts do not. Composite plaster–synthetic casts have not been thoroughly evaluated. This study analyzed the temperature from plaster casts compared with composite casts in a variety of in vitro conditions that would simulate clinical practice.

**Methods:**

A Pyrex cylinder filled with constant body temperature circulating water simulated a human extremity. Circumferential casts, of either plaster or composite construction (plaster inner layer with outer synthetic layer), were applied to the model. Peak temperatures generated by the exothermic reactions were studied relative to the following variables: dip water temperature (24 °C versus 40 °C), cast thickness (16, 30, and 34 ply), and delayed (5-min) versus immediate application of the synthetic outer layers. Peak temperatures from the all-plaster casts were compared with the composite casts of the same thickness. Finally, the relative cast strength was determined.

**Results:**

Potentially dangerous high temperatures were measured only when 40 °C dip water was used or when thick (30- or 34-ply) casts were made. Cast strength increased with increasing cast thickness. However, the presence of synthetics in the composite casts layers did not increase cast strength in every case.

**Conclusion:**

When applying composite casts, the outer synthetic layers should be applied several minutes after the plaster to minimize temperature rise. Composite casts do not routinely generate peak temperatures higher than plaster casts of similar thickness. Because the skin of children and the elderly is more temperature-sensitive than average adult skin, extra care should be taken to limit the exothermic reaction when casting children and the elderly: clean, room temperature dip water, minimal required cast thickness, avoidance of insulating pillows/blankets while the cast is drying.

## Introduction

**Fig. 1:**
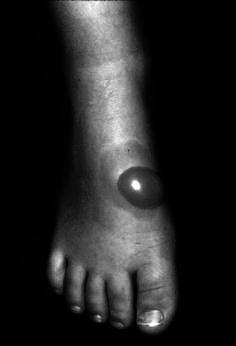
Blister (second-degree burn) at the dorsal aspect of the ankle where the cast is thickest caused by a synthetic-plaster composite cast

Composite walking casts are sometimes used in children for their improved strength and durability. The plaster is applied as the inner layer for its superior moldability; the synthetic is applied as the outer layer for its superior strength and wear characteristics. We observed a second degree burn over the dorsum of the foot in a young child treated with a plaster–synthetic composite cast for idiopathic toe-walking (Fig. 1). We presumed that adding the layer of synthetic material acts as an insulator, increasing the risk of burns. The setting temperatures of both plaster casts and synthetic casts have been previously reported [1, 2]. In these studies a glass tube was used to simulate the extremity; then plaster or synthetic casts were applied, and the peak temperatures were measured.

We measured the temperature from plaster casts compared with composite casts in a variety of conditions that would simulate clinic practice. The variabilities analyzed in different combinations were dip water temperature, cast thickness, and delayed versus immediate application of the synthetic plies. The goal was to determine what combination leads to burn threshold.

## Materials and methods

We applied a total of 120 casts composed of 4-inch J & J Extra-Fast plaster (Johnson & Johnson, Longhorne, PA, USA) or 4-inch J & J Extra-Fast plaster in combination with 4-inch 3M Scotch (3M, St. Paul, MN, USA) casting tape to a Pyrex tube. The variables altered between trials were cast thickness, cast composition (plaster versus plaster and synthetic composite), dip water temperature, and the time interval before applying the Scotch cast outer layer of the composite casts (Table 1, “Appendix”). We used either five or ten casts for each trial. In every trial, a standard polyester-filled hospital pillow was placed over the cast, centered over the thermometer, to simulate the insulating effect of a patient’s leg resting on a pillow.

**Table 1:** Specifications on trials performed

Trial number	Thickness (total)	Plies plaster	Plies of Scotchcast	H_2_O temp (°C)	Scotchcast applied immediately/5 min	Number of casts
1	16	16	–	40	–	10
2	16	16	–	24	–	5
3	16	12	4	40	Immediately	10
4	16	12	4	40	After 5 min	10
5	16	12	4	24	Immediately	5
6	16	12	4	24	After 5 min	5
7	30	30	–	40	–	10
8	30	30	–	24	–	5
9	30	26	4	40	Immediately	10
10	30	26	4	40	After 5 min	10
11	30	26	4	24	Immediately	5
12	30	26	4	24	After 5 min	5
13	34	34	–	40	–	5
14	34	34	–	24	–	5
15	34	26	8	40	Immediately	5
16	34	26	8	40	After 5 min	5
17	34	26	8	24	Immediately	5
18	34	26	8	24	After 5 min	5

These different variables were combined to analyze the temperature from plaster casts compared with composite casts in a variety of conditions that would simulate clinical practice to eliminate the question of what combination leads to burn threshold.

**Fig. 2:**
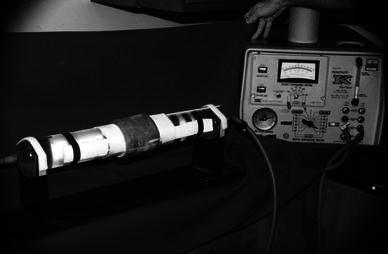
Experimental setup of the Pyrex tube as a limb model

We used a 7.5-cm diameter Pyrex tube 60 cm in length to simulate a patient’s extremity. This tube was connected to a heating unit that provided circulating water at a constant 37.2 ± 0.2 °C (Fig. 2). We attached a mercury thermometer to the superior surface of the Pyrex tube to measure surface (simulated skin) temperature. Cerrobend liquid metal was used to form a secure cap for the thermometer bulb to ensure good thermal contact with the Pyrex surface and eliminate dead air space, which might have acted as insulation. We monitored the water bath temperature by a telethermometer probe, which was placed at the downstream end of the Pyrex tube. The ambient temperature and humidity in the laboratory were monitored throughout the experiment. The room temperature was kept between 19 and 22 °C and the relative humidity was kept at 40–50 %.

Four-inch J & J Extra-Fast plaster (Johnson & Johnson, Longhorne, PA, USA) or 4-inch J & J Extra-Fast plaster in combination with 4-inch 3M Scotch (3M, St. Paul, MN, USA) casting tape was applied to the tube in the following manner: two 6-inch × 1-inch strips of quarter-inch orthopaedic felt were attached to either side of the Pyrex tube to protect the glass from the cast saw used to remove the casts. Next, we wrapped two layers of 6-inch cotton Webril (Fiberweb, Old Hickory, TN, USA) around the tube covering the thermometer and the felt. Clean dip water was used for every cast applied and each roll of casting material was dipped for 10 s. We weighed each roll of material before and after dipping and the increase in weight was maintained between 110 and 140 g to ensure uniform wetting and water content. The casts were applied as 10.2-cm (4-inch) bands centered over the thermometer bulb.

The 16-ply, 30-ply, and 34-ply plaster casts each had 16, 30, and 34 plies of plaster, respectively. For the composite casts of a given ply (16, 30, or 34), four layers of plaster were replaced with synthetic Scotchcast. For example, a 16-ply composite cast was composed of 12 layers of plaster and four layers of synthetic. A 30-ply composite cast had 26 layers of plaster and four layers of synthetic. A 34-ply composite cast had 30 layers of plaster and four layers of synthetic. This was done to simulate the clinical situation in which slightly less plaster is applied when an outer strength wrap of synthetic is planned.

To check the validity of a Pyrex cylinder as a model of an extremity, we applied five casts of 16-ply Extra-Fast plaster to a volunteer’s arm (co-author) in the same manner as used in the Pyrex model tests. The time–temperature curves and peak temperatures generated during these trials were recorded from a temperature probe placed on the volunteer’s skin under the center of the casts. We conducted mechanical tests to determine the relative strength of the various casts studied. The casts from Trials 1, 3, 4, 7, 9, and 10 were cut into 2.5-cm strips, which were then subjected to three-point bending tests. These tests were performed on an Instron 1000 loading machine (Instron, Norwood, MA, USA), and we recorded both the peak load before failure and the time versus load curves.

We regarded the threshold for human skin thermal injury as 49 °C for 4 min. This would be expected to cause a first-degree burn according to Williamson and Scholtz [3]. Surface temperature readings were made at 1-min intervals starting from the time the first roll was dipped. The water bath temperature was also recorded at 1-min intervals and varied by no more than 0.5 °C.

## Results

**Fig. 3:**
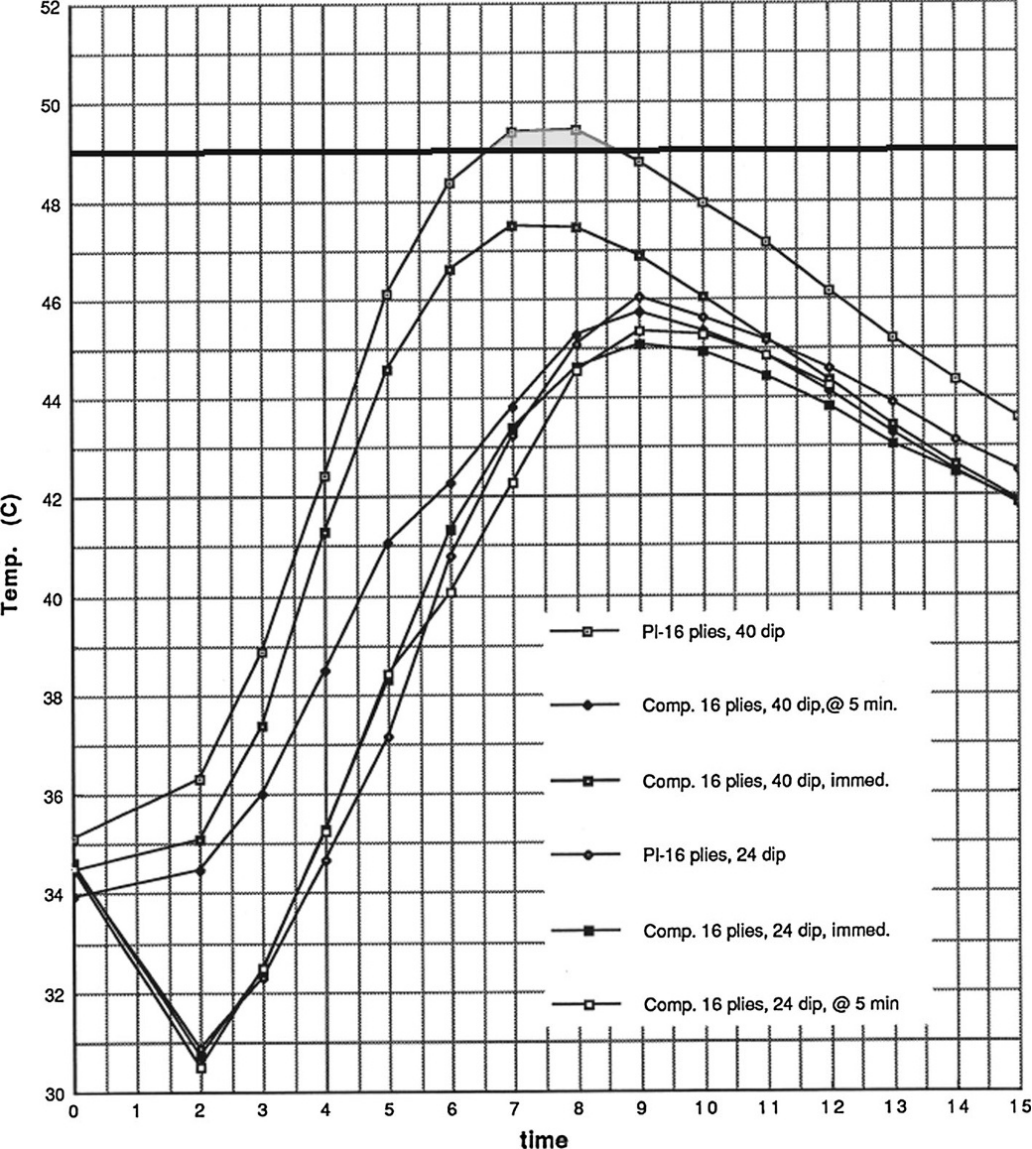
Sixteen-ply plaster and composites. The results are presented as time versus temperature curves. For every trial performed, the mean temperature was calculated at 1-min intervals by averaging the values from the individual castings in a given trial. Thus, for each trial of castings listed in Table 1, a single representative time–temperature curve was generated. These curves are grouped in Figs. 3 through 10 for evaluation and comparison of the effects of dip water temperature, cast thickness, cast type, and delayed versus immediate application of the synthetic outer layer on the peak temperatures and shapes of the time–temperature curves. For clarity, the average values for the specific trials are depicted graphically. The shape of the time–temperature curves in Figs. 3, 4, and 5 are very similar. Figures 3, 4, and 5 also demonstrate the effect of cast thickness on the peak temperatures generated.

**Fig. 4:**
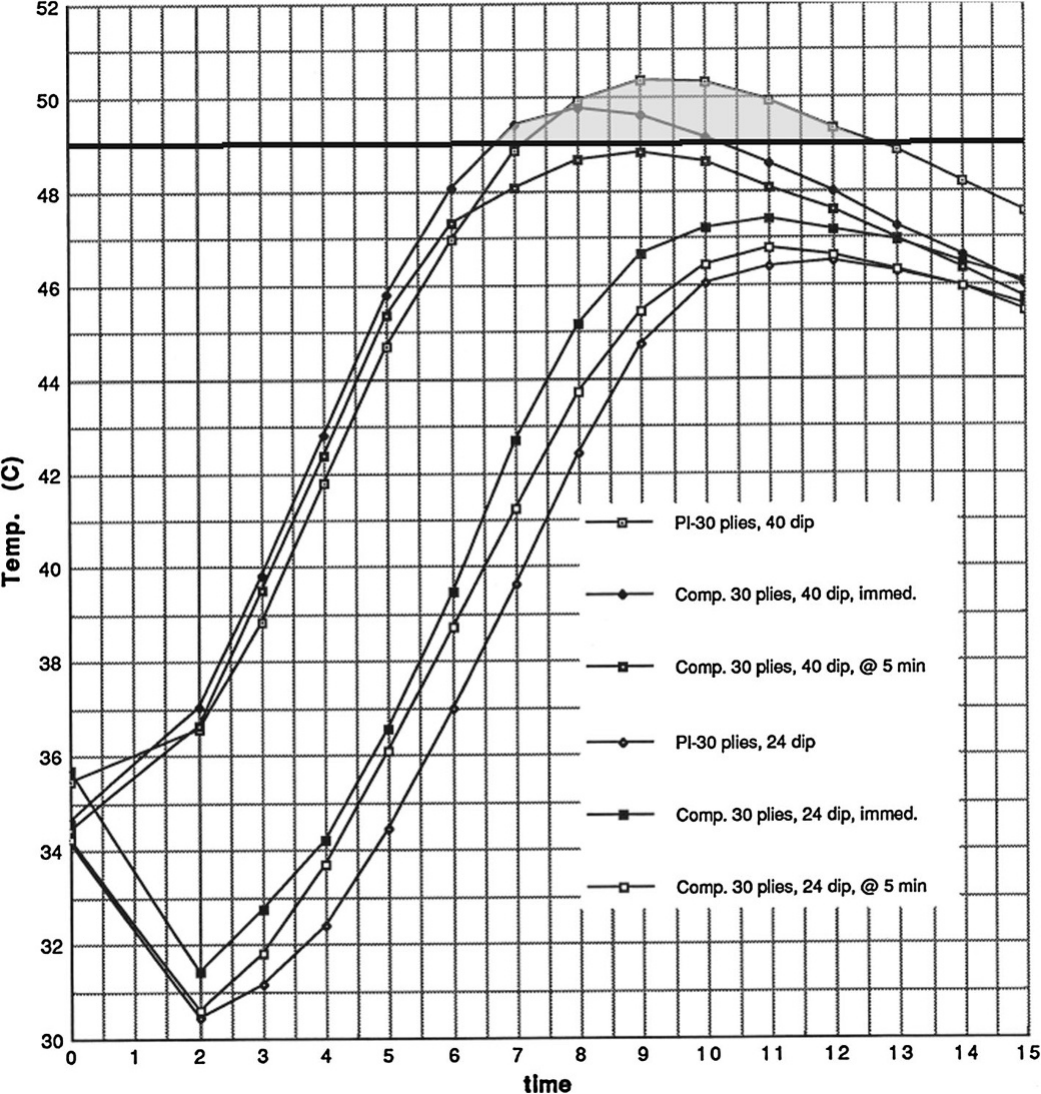
Thirty-ply plaster and composites. The results are presented as time versus temperature curves. For every trial performed, the mean temperature was calculated at 1-min intervals by averaging the values from the individual castings in a given trial. Thus, for each trial of castings listed in Table 1, a single representative time–temperature curve was generated. These curves are grouped in Figs. 3 through 10 for evaluation and comparison of the effects of dip water temperature, cast thickness, cast type, and delayed versus immediate application of the synthetic outer layer on the peak temperatures and shapes of the time–temperature curves. For clarity, the average values for the specific trials are depicted graphically. The shape of the time–temperature curves in Figs. 3, 4, and 5 are very similar. Figures 3, 4, and 5 also demonstrate the effect of cast thickness on the peak temperatures generated.

**Fig. 5:**
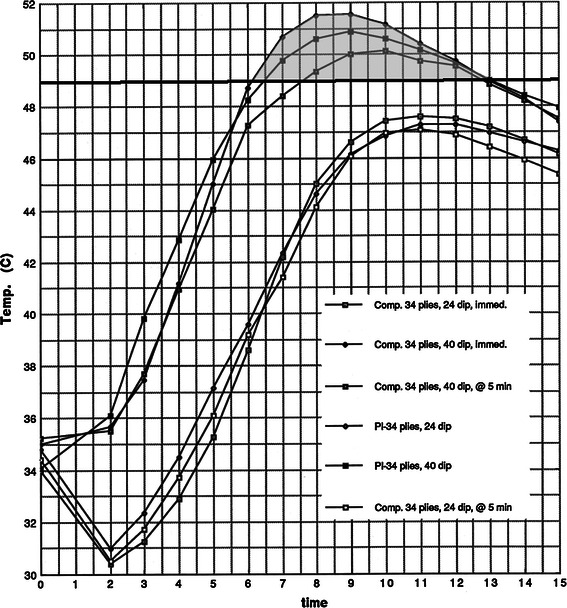
Thirty-four-ply plaster and composites. The results are presented as time versus temperature curves. For every trial performed, the mean temperature was calculated at 1-min intervals by averaging the values from the individual castings in a given trial. Thus, for each trial of castings listed in Table 1, a single representative time–temperature curve was generated. These curves are grouped in Figs. 3 through 10 for evaluation and comparison of the effects of dip water temperature, cast thickness, cast type, and delayed versus immediate application of the synthetic outer layer on the peak temperatures and shapes of the time–temperature curves. For clarity, the average values for the specific trials are depicted graphically. The shape of the time–temperature curves in Figs. 3, 4, and 5 are very similar. Figures 3, 4, and 5 also demonstrate the effect of cast thickness on the peak temperatures generated

In trials with equally thick casts, 40 °C dip water produced peak temperatures 2–4 °C hotter than when 24 °C dip water was used (Figs. 3, 4, 5). The 40 °C dip water trials tended to reach peak temperatures approximately 2 min earlier, but the overall shape of the curves is not noticeably different (Figs. 3, 4, 5).

The peak temperature was directly proportional to the thickness of the cast. Varying the thickness from 16 to 34 plies caused increases in the peak temperatures in the range of 1–4 °C. Increasing the number of plies also tends to broaden the time–temperature curve slightly.

When all the 16-ply casts were compared, only the plaster/40 °C dip water cast reached burn threshold (Fig. 3). In the 30-ply trials, burn threshold was reached in the plaster/40 °C and the composite/40 °C/immediate casts. Waiting 5 min (composite/40 °C/5 min) apparently allowed enough dissipation of heat to prevent burns (Fig. 4). In the 34-ply trials, burn threshold was reached in all cases when 40 °C dip water was used. The 5-min delay was not enough to be protective in these thick casts (Fig. 5).

**Fig. 6:**
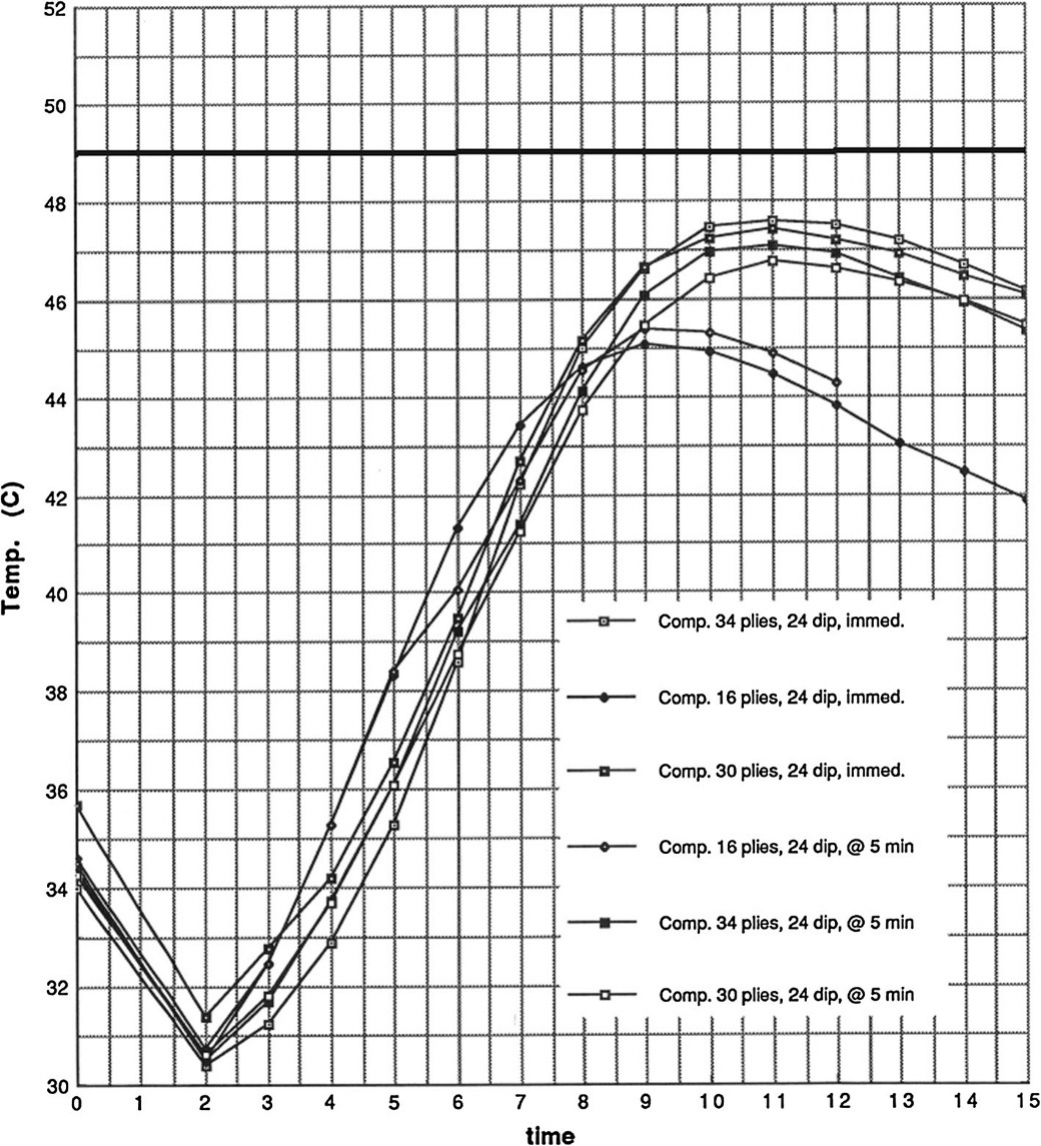
Composites, 24 °C; delayed versus immediate application of the synthetic layers. The results are presented as time versus temperature curves. For every trial performed, the mean temperature was calculated at 1-min intervals by averaging the values from the individual castings in a given trial. Thus, for each trial of castings listed in Table 1, a single representative time–temperature curve was generated. These curves are grouped in Figs. 3 through 10 for evaluation and comparison of the effects of dip water temperature, cast thickness, cast type, and delayed versus immediate application of the synthetic outer layer on the peak temperatures and shapes of the time–temperature curves. For clarity, the average values for the specific trials are depicted graphically. The shape of the time–temperature curves in Figs. 3, 4, and 5 are very similar. Figures 3, 4, and 5 also demonstrate the effect of cast thickness on the peak temperatures generated.

**Fig. 7:**
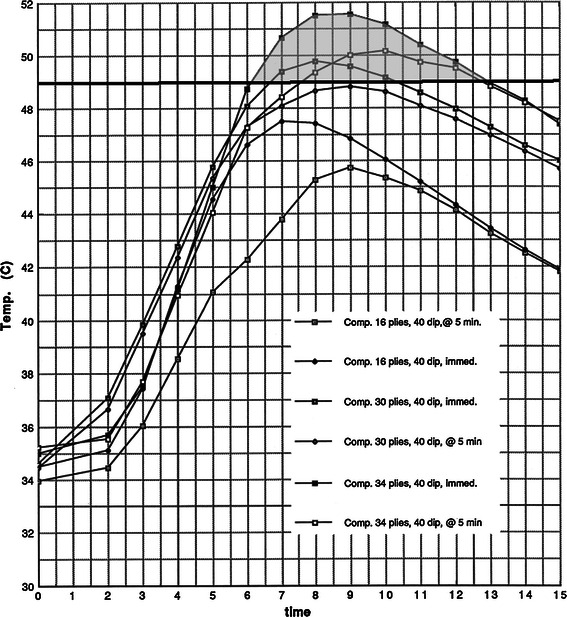
Composites, 40 °C; delayed versus immediate application. The results are presented as time versus temperature curves. For every trial performed, the mean temperature was calculated at 1-min intervals by averaging the values from the individual castings in a given trial. Thus, for each trial of castings listed in Table 1, a single representative time–temperature curve was generated. These curves are grouped in Figs. 3 through 10 for evaluation and comparison of the effects of dip water temperature, cast thickness, cast type, and delayed versus immediate application of the synthetic outer layer on the peak temperatures and shapes of the time–temperature curves. For clarity, the average values for the specific trials are depicted graphically. The shape of the time–temperature curves in Figs. 3, 4, and 5 are very similar. Figures 3, 4, and 5 also demonstrate the effect of cast thickness on the peak temperatures generated.

Regarding the effects of delayed (5-min) versus immediate application of the synthetic plies in the composite cast trials, there was very little variation in peak temperatures when 24 °C dip water was used (Fig. 6). However, there was more variation when 40 °C dip water was used (Fig. 7). An increase in peak temperatures of 1–2 °C was observed when the synthetic casting material was added immediately. In addition, the curves in the 40 °C trials are widened and shifted slightly to the left, indicating greater overall exothermic reaction. Adding the synthetic layers immediately increased the peak temperatures and lengthened the time these temperatures were maintained. In the 40 °C dip water trials, burn threshold was reached in the 34-ply/composite/immediate, the 34-ply/composite/5-min delay, and the 30-ply/composite/immediate trials (Fig. 7).

The time–temperature curves and peak temperatures were within ±1.0 °C of the temperatures recorded from the Pyrex model encased in the same 16-ply Extra-Fast plaster cast.

**Fig. 8:**
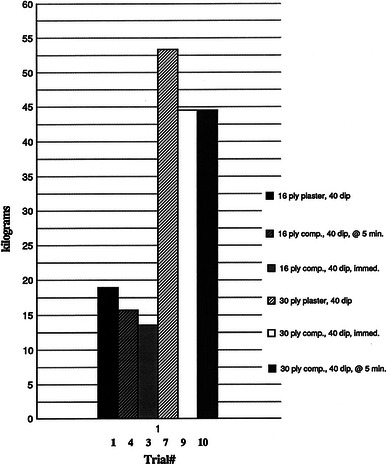
Results of the three-point bending tests

The average peak force required to deform the 2.5 × 10.2-cm strips of casting material from Trials 1, 3, 4, 7, 9, and 10 varied as would be expected. Increasing cast thickness increased the strength of the material tested. However, the presence of the synthetic layers on the composite casts (Trials 3, 4, 9, and 10) did not appear to increase the strength of the material in these tests. We observed no significant difference (*p* > 0.05) between the 30-ply trials (7, 9, and 10) or between Trials 1 and 4. There was, however, a significant difference (*p* < 0.05) between Trials 1 and 3, the 16-ply casts were significantly stronger than the combination of 12 plies of cast and 4 plies of synthetic cast immediately applied (Fig. 8).

## Discussion

**Fig. 9:**
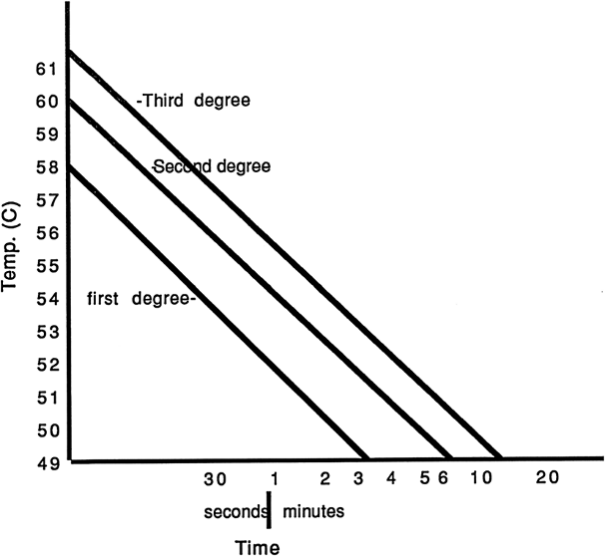
Type of burn as a function of temperature and time (after Williamson and Scholtz^11^)

The exothermic reaction of cast material is a well known problem. Plaster of paris as well as synthetic casts have been analyzed; however, composite plaster–synthetic cast have never been extensively tested. Previous studies attempted to define the time–temperature relationship in the production of epidermal burns [3–9]. As temperature of the thermal insult is increased, the time required to produce a specific injury is decreased substantially (Fig. 9). For example, with a temperature of 50 °C, approximately 5 min of exposure is required to cause a second-degree burn, whereas only 1 min of exposure is needed at 54 °C. Elderly people and young children tend to be more susceptible to burn injury because of decreased skin thickness, decreased reaction time, or decreased cutaneous sensation [10].

Burns from orthopaedic plaster cast splints, although not common occurrences, have been reported [5, 6, 11]. Previous investigators have evaluated the exothermic properties of plaster or synthetic casting materials separately. Ganaway and Hunter [12] and Lavalette and Pope [1] demonstrated plaster could, under the right set of conditions, create temperatures at or above the burn threshold. Synthetic materials, however, have a smaller exothermic potential and even under the worst conditions did not create dangerous temperatures [2]. The important variables identified in previous studies which influenced the temperatures generated have been cast thickness, dip water temperature, type of casting material, and the presence of an insulator (pillow) [1–7, 9, 12, 13]. Deignan et al. [14] were even able to show that pressure applied during themolding of casts results in a significant increase in temperature at the side where the mold was applied. Variables of lesser importance included the amount of cast padding, the room humidity, the room temperature, and the use of previously used dip water. Haasch [5] emphasized that when the cast rolls are soaked in hot water, they absorb water at a slower rate than when they are soaked in cold water. Also, if the cast rolls are dipped in water for a short period of time, they absorb less water. When less water is present in the cast rolls, the temperature of the cast increases. The specific heat capacity of water of 4,190 J/kg K is very high, which means the more water is present, the more of a cooling effect the water has on the temperature of the cast.

We asked whether, under identical conditions, composite casts would produce greater skin surface temperatures than plain plaster casts. We found neither plaster nor composite casts were consistently hotter. These data indicate when dip water temperature, cast thickness, and all other variables are controlled, composite casts present no greater threat of causing thermal injury than pure plaster casts. However, composite casts are more likely to cause thermal injury than similar pure synthetic casts. Dip water temperature seems to be important. At no time, regardless of cast thickness, did we measure a “skin” surface temperature >48 °C when 24 °C dip water was used. When 40 °C dip water was used, our thinnest plaster cast (16-ply) approached burn threshold (49.5 °C). We suggest the dip water should feel slightly cool to the touch. The 40 °C dip water we used felt pleasantly warm. Water that feels warm should not be used because of the increased risk of thermal injury.

Our data regarding cast thickness were consistent with previous research. To reduce the risk of burns, it is prudent to limit the amount of casting material, provided it can be accomplished without compromising cast strength. In addition to measuring the thickness of every cast we made, we measured the thickness of 12 short leg and long leg casts selected at random from the cast room of our clinic. The dorsum of the ankle was consistently the thickest area because its relatively shorter radius of curvature when compared with the heel created more overlapping of the plies. The average thickness at this location was 11.6 ± 1.6 mm. This value compares reasonably well with our experimental casts, which measured 7–8 mm for 16 plies, 12–13 mm for 30 plies, and 13–14 mm for 34 plies. Therefore, our experimental casts were fairly representative of what might be used in clinical practice.

The final variable we analyzed was the time at which we applied the synthetic layer to our composite casts. Although it was not as important a factor as dip water temperature or cast thickness, we found the exothermic response could be lessened by 1–2 °C if the synthetic layer was added after a short (5-min) delay.

The individual castings produced time–temperature curves that were nearly identical within each of the 18 trial groups (Table 1, “Appendix”). At no time did we measure peak temperatures that varied by more than ±1.0 °C within any single trial. The peak temperatures reported by Lavalette and Pope [1] of 63–68 °C are considerably higher than the temperatures recorded under similar conditions. These differences might, in part, be a result of the different experimental designs used. Ganaway and Hunter [12] used an experimental setup very similar to our own. Their results are much more consistent with the temperatures we have reported. As mentioned previously, we performed five casts on a volunteer’s forearm under the same conditions as in Trial 2. The peak temperatures did not vary by more than ±1.0 °C between these trials. For this and the previously stated reasons, we believe our data were consistent, reproducible, and a reasonable approximation of what might be expected in clinical practice. One potential weakness of the study is specific formulations of plaster or synthetics may vary from manufacturer to manufacturer.

The three-point bending tests performed on selected casts (Fig. 8) revealed no major difference in material strength between the 16-ply trials and the 30-ply trials. Callahan et al. [15, 16] recommended three-point bending and diametric compression tests to best characterize the material strength of casts. In the present study, we performed three-point bending tests. From our data, it appears the composite material is no stronger than an equal number of plies of plaster. The clinical importance of these data is unclear, because the materials were not tested in the form of intact, cylindrical casts nor were they tested for scuff resistance or torsional stability.

Based on our data, we concluded the temperature of the dip water was the most important variable in generating heat in composite casts. At no time was a peak temperature greater than 48 °C recorded when 24 °C dip water was used. When applying composite casts, it may be prudent to delay the addition of the outer synthetic layers for several minutes to minimize the peak temperature generated. There appears to be no increased risk of thermal injury with composite casts compared with equally thick plaster casts. Because there is a wide range of susceptibility to thermal injury, it is advisable to take steps to minimize the exothermic reaction at all times. Using cool dip water and applying the minimum necessary amount of plaster casting material should help avoid the problem of exothermic burns. It is possible that increased thickness of padding under the cast may be protective, with the trade-off of a less well fitting cast.

Plaster of Paris casts are used extensively around the world. Synthetic casts, although more expensive, are used more commonly in wealthier parts of the world. Many pediatric orthopaedists prefer plaster because of its perceived superior moldability [17]. The revival of the Ponseti method has stimulated an increased use of plaster casts, because this is the material of choice recommended by Ponseti [17]. In older children undergoing casting, especially after walking age, plaster casts may break or degrade from scuffing. Adding a layer of synthetic cast improves durability while still allowing the superior moldability of plaster of Paris.

One weakness of our study is that our experimental model of a glass tube is not the best currently available model to study the thermal effects of casts. Recent experimental studies by Halanski et al. [6] have used a different model that more accurately mimics the human skin–cast interface: PVC tubing layered with a carbon fibre heating element. Our model, with its increased thermal conductivity of the glass tube model, may tend to underestimate the risk of burns, as the glass would conduct the heat away from the skin thermocouples. This may explain why very few of our trial results approached the burn threshold. Therefore, we advise all health care professionals applying casts to utilize all available means to minimize the risk of thermal injury when applying plaster or composite casts to children or adults: room temperature, clean, dip water; minimum required thickness of plaster; avoid covering the cast with blankets while it is drying; avoid setting the freshly applied cast on an insulating pillow. These guidelines and recommendations have been emphasized by other authors, most recently in an excellent review article by Halanski and Noonan [18].

## Appendix

See Table 1
